# Pregnancy Outcomes of Single/Double Blastocysts and Cleavage Embryo Transfers: a Retrospective Cohort Study of 24,422 Frozen-Thawed Cycles

**DOI:** 10.1007/s43032-020-00247-x

**Published:** 2020-08-25

**Authors:** Xiaoyu Long, Yuanyuan Wang, Fangrong Wu, Rong Li, Lixue Chen, Weiping Qian, Jie Qiao

**Affiliations:** 1 Center for Reproductive Medicine, Department of Obstetrics and Gynecology, Peking University Third Hospital, No.49 North Huayuan Road, Haidian District, Beijing, 100191 China; 2 National Clinical Research Center for Obstetrics and Gynecology, Beijing, 100191 China; 3 Department of Reproductive Medicine, Peking University Shenzhen Hospital, Shenzhen, Guangdong China

**Keywords:** Transfer strategy, Double-cleavage embryo transfer, Single-blastocyst embryo transfer

## Abstract

This study aims to evaluate the effect of blastocyst- and cleavage-stage embryo transfers with different numbers of transferred embryos on pregnancy outcomes in China. This was a retrospective cohort study that collected 24,422 frozen-thawed embryo transfer (FET) cycles in two affiliated hospitals of Peking University Health Science Center between January 2015 and May 2018. They were divided into four groups: the single cleavage-stage embryo transfer group (C-1) (763 cycles), double cleavage-stage embryo transfer group (C-2) (13,004 cycles), single blastocyst-stage embryo transfer group (B-1) (7913 cycles), and double blastocyst-stage embryo transfer group (B-2) (2046 cycles). Of the four groups, the live birth rate was the lowest in the C-1 group (11.8%) while it was the highest in the B-2 group (33.6%). However, the B-2 group was accompanied with higher risks of miscarriages, maternal complications, twin births, preterm births, and low birth weight. Compared with the C-2 group, the B-1 group had a lower live birth rate (23.0 vs 29.0%; aOR, 0.78; 95% CI, 0.72–0.85), but also had a lower risk for twin births (1.9 vs 23.4%; aOR, 0.06; 95% CI, 0.04–0.09) and preterm births (9.6 vs 16.1%; aOR, 0.51; 95% CI, 0.41–0.65). The probability of live birth in the B-1 group declined from 0.25 at 20–29 years old to 0.08 at > 40 years old, while the probabilities of adverse outcomes went up with maternal age. It can be concluded that **s**ingle-blastocyst embryo transfer seems to be the best choice for all maternal ages. This group of embryo transfer has significantly reduced adverse neonatal outcomes. Especially, women with younger maternal age in this group appear to prominently benefit from single-blastocyst transfer.

**Electronic supplementary material:**

The online version of this article (10.1007/s43032-020-00247-x) contains supplementary material, which is available to authorized users.

## Introduction

It has been over 40 years since the development of assisted reproductive technology. With improvements such as ovulation induction, embryo culture, vitrification, and other technologies, experts in the field of reproductive medicine are no longer only concerned about pregnancy rates, but also healthy live births.

At present, common clinical transfer strategies include double-cleavage embryo transfer and single-blastocyst embryo transfer. In addition, single-cleavage embryo transfer and double-blastocyst embryo transfers have been commonly performed in the clinic. Previous studies demonstrated that women who undergo fresh blastocyst transfers achieve higher live-birth rates compared with those who receive fresh cleavage-stage transfers [1]. For the thawing transfer cycle, the results are not quite so conclusive [2]. Studies have shown that the rates of clinical pregnancy and ongoing pregnancy of human embryos that were vitrified and thawed at the blastocyst stage were significantly higher than that at cleavage stages [3, 4]. However, other studies have proposed that single-blastocyst embryo transfer could increase the implantation rate but has the same live birth rate as double-cleavage embryo transfer. Also, there are different opinions on the perinatal outcome of blastocyst embryo transfer and cleavage embryo transfer. Some studies have found that perinatal mortality and risk of placental complications were higher in the blastocyst group as compared to the cleavage-stage group [5, 6]. However, another study pointed out that obstetric and perinatal outcomes after blastocyst transfer were similar when compared with embryo cleavage-stage transfers [7, 8].

We aimed to individually evaluate the effect of blastocyst and cleavage embryo transfer number on pregnancy outcomes. We selected two large reproductive medical centers, one in Beijing and the other in Shenzhen, to represent the northern and southern regions of China, respectively. The purpose of this study is to guide clinicians to choose the most suitable transfer strategy by analyzing the pregnancy outcomes of four different transfer strategies.

## Materials and Methods

### Data Source and Variables

This was a retrospective cohort study that used the ART databases from two affiliated hospitals of Peking University (Peking University Third Hospital and Peking University Shenzhen Hospital). 24,422 frozen-thawed embryo transfer (FET) cycles recorded from January 2015 to May 2018 were selected to generate the research database. The database consisted of the following information: maternal characteristics (maternal age at treatment, height, weight, infertility type, cause of infertility and duration of infertility, and number of previous ART cycles), treatment records of the current cycle (type of fertilization in the current cycle, numbers of embryo transferred, days of embryo development), maternal outcomes (clinical pregnancy, monozygotic twins, miscarriages, and maternal complications), and neonatal outcomes (live births, number of live births, gestational age at delivery, birth weight, and congenital malformations).

**Fig. 1:**
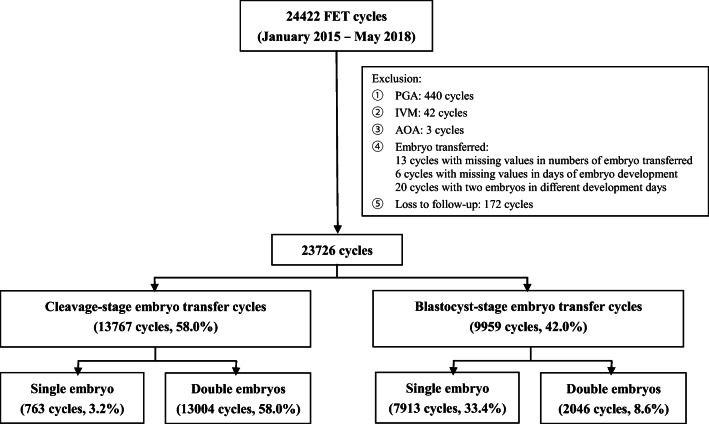
Flow chart showing the data selection process for analysis in this study

Of the 24,422 FET cycles, 440 preimplantation genetic analysis (PGA) cycles, 42 in vitro maturation (IVM) cycles, 3 assisted oocyte activation (AOA) cycles, 13 cycles with missing values for the number of embryos transferred, 6 cycles with missing data for days of embryo development, 20 cycles with two embryos in different development days, and 172 cycles with loss to follow-up, were excluded from the analysis. Finally, 23,726 cycles were included for the final analysis. This included 13,767 cleavage-stage embryo transfer cycles (58.0%) and 9959 blastocyst-stage embryo transfer cycles (42.0%). Based on the stage (cleavage stage or blastocyst stage) and number (one or two) of embryos transferred, all the selected cycles were divided into four exposure groups: the single cleavage-stage embryo transfer group (C-1) (763 cycles, 3.2%), double cleavage-stage embryo transfer group (C-2) (13,004 cycles, 58.0%), single blastocyst-stage embryo transfer group (B-1) (7913 cycles, 33.4%), and double blastocyst-stage embryo transfer group (B-2) (2046 cycles, 8.6%) (Fig. 1).

The following data were analyzed for each cycle: maternal age (20–29 years, 30–34 years, 35–37 years, 38–39 years, 40–42 years, or > 42 years), body mass index (BMI) (equal to height/weight2 (m/kg^2^), BMI < 18.5 defined as “underweight,” 18.5 ≤ BMI < 24 defined as “normal weight,” 24 ≤ BMI < 27 defined as “overweight,” or BMI ≥ 27 defined as “obesity”), infertility type (primary infertility, or secondary infertility), cause of infertility (tubal, ovulatory, endometriosis, male factor, or unexplained), duration of infertility (≤ 4 years or > 4 years), type of fertilization in the current cycle (IVF, ICSI, or IVF + ICSI), previous ART cycles (0–1 cycles, 2–3 cycles, 4–5 cycles, or ≥ 6 cycles), clinical pregnancy (yes or no), monozygotic twins (yes or no), miscarriage (yes or no), maternal complications (yes or no, including hypertensive disorders, diabetes, thyroid diseases, placental complications, postpartum hemorrhage, and premature rupture of membranes), live birth (yes or no), twin birth (yes or no), preterm birth (defined as “gestational week at delivery < 37 weeks,” yes or no), low birth weight (defined as “birth weight at delivery < 2500 grams,” yes or no), small for gestational age infant (SGA, defined as “birth weight at delivery < 10th percentile for the baby’s gender and gestational age, using Chinese reference charts” [9], yes or no), and congenital malformations (yes or no). With regard to cycles with multiple pregnancies or births, the cycle was defined as “yes” for live births, preterm births, low birth weights, SGA, or congenital malformations.

The study was approved by the Peking University Third Hospital Medical Science Research Ethics Committee (IRB00006761-M2019107).

### Statistical Analysis

Comparisons among the four groups or between any two groups were performed using the χ2 test (Pearson chi-square for cases where none of the cells has expected counts less than 5; Likelihood ratio for cases where one or more cells had expected counts less than 5). *P* < 0.05 was considered statistically significant for comparisons among all four groups and *P* < 0.008 was considered statistically significant for comparisons between any two groups. The Bonferroni-corrected *P* value was used for multiple pairwise comparisons (the alpha-level was divided by the number of pairwise comparisons, the number of pairwise comparisons for all four groups was 6, i.e., equal to 0.05/6).

In addition, comparisons between the C-2 group and the B-1 group were performed. Unadjusted logistic regressions were initially performed to calculate unadjusted odds ratios (ORs) and 95% confidence intervals (CIs) of the B-1 group vs the C-2 group for each maternal or neonatal outcome. Multivariable logistic regressions were then performed to calculate adjusted odds ratios (aORs) and 95% CIs, which included maternal age, BMI, infertility type, cause of infertility, duration of infertility, type of fertilization in the current cycle, and previous ART cycles. Furthermore, multivariable logistic regression models were used to predict absolute risks (probabilities) for each maternal or neonatal outcome for each maternal age group. This was graphically presented to illustrate age-outcome relationships.

All analyses were performed using IBM SPSS Statistics 25.0 (Armonk, NY: IBM Corp.).

## Results

### Maternal Characteristics

A total of 23,726 FET cycles were included in this study, of which, 763 cycles were single cleavage-stage embryo transfers (C-1 group), 13,004 were double cleavage-stage embryo transfers (C-2 group), 7913 were single blastocyst-stage embryo transfers (B-1 group), and 2046 were double blastocyst-stage embryo transfers (B-2 group). Maternal characteristics among the four groups were statistically different for maternal age, BMI, infertility type, cause of infertility, duration of infertility, type of fertilization in the current cycle, and previous ART cycles (*P* < 0.05) (Supplementary 1). Results of pairwise comparisons of maternal characteristics between the groups are shown in Supplementary 2.

### Maternal and Neonatal Outcomes for the Different Transfer Strategies

**Fig. 2:**
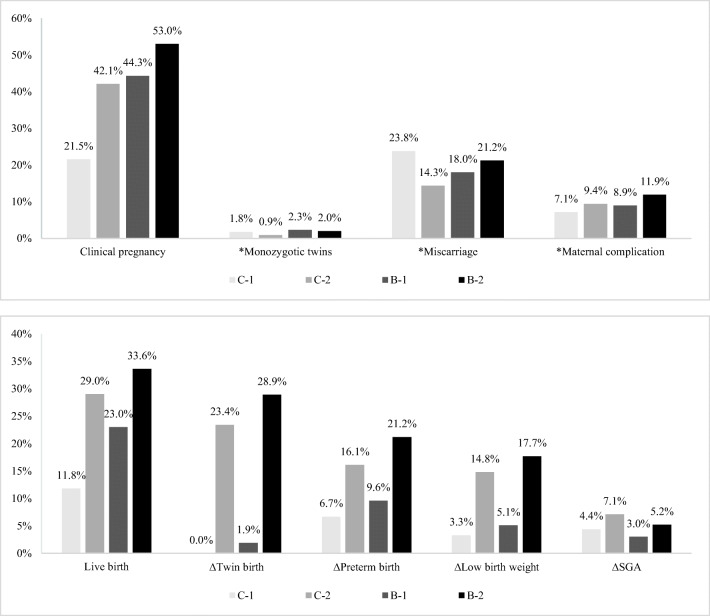
Percentages of each maternal or neonatal outcome in each group. C-1 indicates the single cleavage-stage embryo transfer group; C-2 indicates the double cleavage-stage embryo transfer group; B-1 indicates the single blastocyst-stage embryo transfer group; B-2 indicates the double blastocyst-stage embryo transfer group. * The denominator is the number of clinical pregnancies in each group. ∆ The denominator is the number of live births in each group

Maternal and neonatal outcomes for all FET cycles and for each group are summarized in Fig. 2. Maternal and neonatal outcomes for the four groups were statistically different for clinical pregnancy, monozygotic twins, miscarriages, maternal complications, live births, twin births, preterm births, low birth weight, and SGA (*P* < 0.05) (Supplementary 1).

Results of pairwise comparisons for maternal and neonatal outcomes between the groups are shown in Supplementary 2 (*P* < 0.008 was considered statistically significant for multiple pairwise comparisons). Among the four groups, the clinical pregnancy rate and live birth rate were the lowest in the C-1 group (21.5% and 11.8%, respectively) and the highest in the B-2 group (53.0% and 33.6%, respectively). However, the B-2 group was accompanied with higher risks of miscarriages (21.2%), maternal complications (11.9%), twin births (28.9%), preterm births (21.2%), and low birth weight (17.7%) compared to the other three groups. These results demonstrated that the double cleavage-stage embryo transfer (C2) strategy and the single blastocyst-stage embryo transfer (B1) strategy were preferred among the four groups. Hence, additional comparisons between the C-2 and B-1 groups were performed.

In this study, 20 cases (0.3%) of congenital malformations were reported for live births and included one case (1.1%) in the C-1 group, 12 cases (0.3%) in the C-2 group, 6 cases (0.3%) in the B-1 group, and one case (0.1%) in the B-2 group. However, due to the limited sample size, comparisons of congenital malformations between the groups were not performed.

### Comparison Between Double-Cleavage Transfer and Single-Blastocyst Transfer

After adjusting for maternal age, BMI, infertility type, cause of infertility, duration of infertility, type of fertilization in the current cycle, and previous ART cycles (Table 1), the clinical pregnancy rates between two groups were not statistically different (B-1 vs C-2, 44.3 vs 42.1%; aOR 1.04; 95% CI, 0.97–1.12). The live birth rate in the B-1 group was lower compared to the C-2 group (23.0 vs 29.0%; aOR, 0.78; 95% CI, 0.72–0.85). With regard to negative outcomes, compared to the C-2 group, the B-1 group had a higher risk for monozygotic twins (2.3 vs 0.9%; aOR, 3.02; 95% CI, 1.79–5.10) and miscarriages (18.0 vs 14.3%; aOR, 1.29; 95% CI, 1.11–1.51), but had a lower risk for twin births (1.9 vs 23.4%; aOR, 0.06; 95% CI, 0.04–0.09), preterm births (9.6 vs 16.1%; aOR, 0.51; 95% CI, 0.41–0.65), low birth weights (5.1 vs 14.8%; aOR, 0.30; 95% CI, 0.22–0.40), and SGA (3.0 vs 7.1%; aOR, 0.51; 95% CI, 0.33–0.77).

**Table 1:** Comparison of maternal and neonatal outcomes between the double-cleavage transfer group (C-2) and the single-blastocyst transfer group (B-1)

	C-2 (*n* = 13,004)	B-1 (*n* = 7913)	B-1 vs C-2	
			OR (95% CI)	aOR (95% CI)
Clinical pregnancy	5481 (42.1%)	3507 (44.3%)	1.09 (1.03, 1.16)	1.04 (0.97, 1.12)
*Monozygotic twins	47 (0.9%)	79 (2.3%)	2.66 (1.85, 3.83)	3.02 (1.79, 5.10)
*Miscarriage	786 (14.3%)	632 (18.0%)	1.31 (1.17, 1.47)	1.29 (1.11, 1.51)
*Maternal complication	515 (9.4%)	312 (8.9%)	1.25 (1.07, 1.46)	0.89 (0.74, 1.06)
Live birth	3771 (29.0%)	1819 (23.0%)	0.73 (0.69, 0.78)	0.78 (0.72, 0.85)
^∆^Twin birth	883 (23.4%)	34 (1.9%)	0.06 (0.04, 0.09)	0.06 (0.04, 0.09)
^∆^Preterm birth	609 (16.1%)	175 (9.6%)	0.55 (0.46, 0.66)	0.51 (0.41, 0.65)
^∆^Low birth weight	559 (14.8%)	92 (5.1%)	0.31 (0.24, 0.39)	0.30 (0.22, 0.40)
^∆^SGA	267 (7.1%)	55 (3.0%)	0.41 (0.30, 0.55)	0.51 (0.33, 0.77)

C-2 indicates the double cleavage-stage embryo transfer group; B-1 indicates the single blastocyst-stage embryo transfer group. * The denominator is the number of clinical pregnancies in each group. ^∆^ The denominator is the number of live births in each group. OR is the unadjusted odds ratio for each maternal or neonatal outcome; aOR is adjusted for maternal age, BMI, infertility type, cause of infertility, duration of infertility, type of fertilization in current cycle, and previous ART cycles

**Fig. 3:**
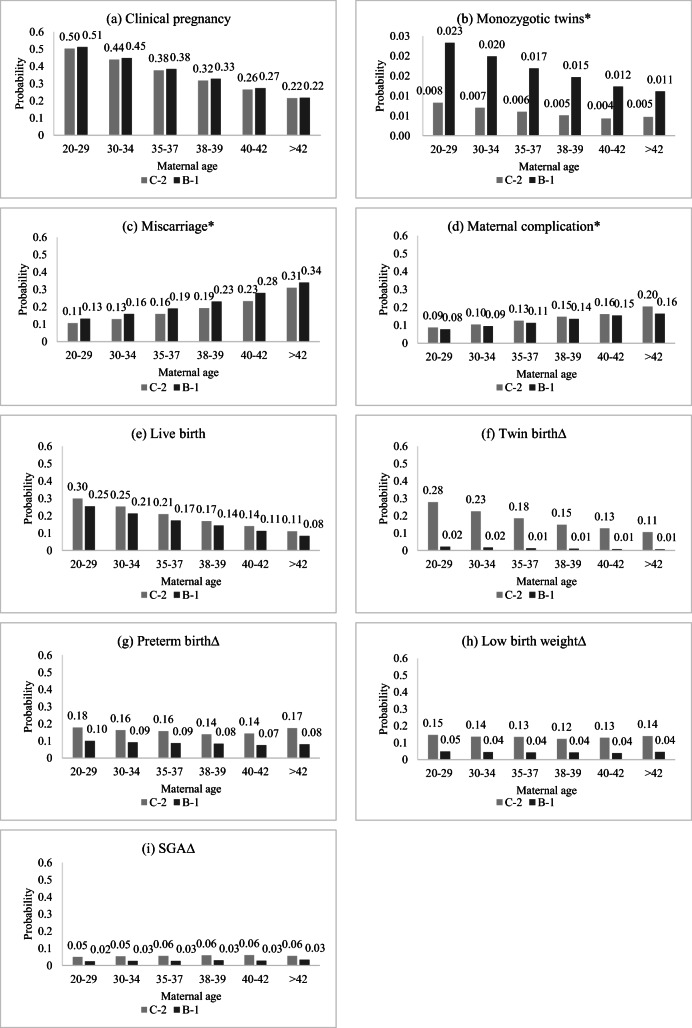
Probabilities of each maternal or neonatal outcome in each maternal age group. C-2 indicates the double cleavage-stage embryo transfer group; B-1 indicates the single blastocyst-stage embryo transfer group. * The denominator is the number of clinical pregnancies in each group. ∆ The denominator is the number of live births in each group. The probabilities of each maternal or neonatal outcome in each maternal age group were calculated by using the model of multivariable logistic regressions

Maternal age is often considered as one of the most important determinants for maternal and neonatal outcomes for any type of ART cycle. Therefore, maternal or neonatal outcomes between the C-2 group and B-1 group and probabilities for each maternal age group (20–29 years old, 30–34 years old, 35–37 years old, 38–39 years old, 40–42 years old, and > 42 years old) were calculated. For both groups, the probabilities of clinical pregnancy and live birth, as well as monozygotic twins and twin births, went down with increased maternal age, while the probabilities of miscarriage and maternal complications went up with increased maternal age. Results are shown in Fig. 3 (detailed results are shown in Supplementary 3 and Supplementary 4).

With regard to the C-2 group, the probability of clinical pregnancy declined from 0.50 at 20–29 years old to 0.22 at > 40 years old. Of the cycles with clinical pregnancy, the probability of monozygotic twins decreased from 0.008 to 0.005, the probability of miscarriages increased from 0.11 to 0.31, and the probability of maternal complications increased from 0.09 to 0.20 at the same maternal age intervals. The probability of live birth declined from 0.30 at 20–29 years old to 0.11 at > 40 years old. Of the cycles with live births, the probability of twin births declined from 0.28 to 0.11, and the probabilities of preterm births, low birth weights, and SGA slightly fluctuated from 0.14 to 0.18, 0.12 to 0.15, and 0.05 to 0.06, respectively.

With regard to the B-1 group, the probability of clinical pregnancy declined from 0.51 at 20–29 years old to 0.22 at > 40 years old. Among those cycles with clinical pregnancy, the probability of monozygotic twins decreased from 0.023 to 0.011, the probability of miscarriages increased from 0.13 to 0.34, and the probability of maternal complications increased from 0.08 to 0.16 at the same maternal age intervals. The probability of live birth declined from 0.25 at 20–29 years old to 0.08 at > 40 years old. Of the cycles with live births, the probability of twin births, preterm births, low birth weight, and SGA slightly fluctuated from 0.01 to 0.02, 0.07 to 0.10, 0.04 to 0.05, and 0.02 to 0.03, respectively.

## Discussion

The strategy of performing twin embryo transfers during the IVF-ET procedure is preferred to attain a higher clinical pregnancy rate. However, using this strategy increases the probability of multiple pregnancies [10]. Multiple pregnancies is a serious complication encountered during assisted reproduction. Compared to singleton pregnancies, it can increase the incidence of miscarriages, fetal deaths, fetal malformations, fetal intrauterine growth restrictions, and the incidence of pregnancy complications such as preterm birth, maternal anemia, pregnancy-induced hypertension, gestational diabetes, and postpartum hemorrhage [11–13].

In order to reduce the occurrence of multiple pregnancies, the number of embryos transferred should be reduced. However, this could reduce the pregnancy rate. How best to reduce the number of transferred embryos without affecting the pregnancy rate is important for the success of assisted reproductive technology. Although single-cleavage embryo transfer logically reduces multiple pregnancies, the clinical pregnancy rate and live birth rate of single cleavage embryo transfers are significantly reduced in older women. Hence, single-cleavage embryo transfers are rarely used in clinical practice. Several studies have shown that the implantation rate and clinical pregnancy rate of blastocyst transfer are higher compared to cleavage embryo transfer, while the rate of multiple pregnancies and ovarian hyperstimulation is lower [14, 15]. Previous studies have compared the clinical outcomes of double-cleavage embryo transfer and single-blastocyst embryo transfer. However, these were small cohort studies that were not segregated into the four different transfer groups or segregated based on age [1, 16].

In this study, we analyzed a very large sample cohort derived from two centers in China including four transfer strategies. We then compared their clinical pregnancy rate and perinatal outcome and stratified them based on maternal age. Considering the significant heterogeneity between groups for many parameters of maternal characteristics, we conducted multivariable logistic regression models to reduce the potential interference from these confounding factors. After adjustment, there was no significant difference in the clinical pregnancy rate between the double cleavage-stage embryo transfer group and single blastocyst-stage embryo transfer group, but the twin birth rate, preterm birth rate, and low birth weight rate were significantly increased in the double cleavage-stage embryo transfer group. This is not consistent with our purpose of delivering healthy newborns. In order to reduce the incidence of maternal-fetal complications and multiple pregnancies, the single-blastocyst embryo transfer strategy seems to be the best choice. Blastocyst transfer is more in line with the physiological environment compared to cleavage embryo transfer because under natural physiological conditions, the cleavage embryo develops in the fallopian tube and does not enter the uterine cavity until the blastocyst stage. In addition, endometrium and embryo development are more synchronous and conducive to embryo implantation. At the same time, from the cleavage stage to blastocyst stage, the embryo has undergone a screening, with only good quality embryos developing to the blastocyst stage in vitro.

Maternal age is an important factor for the outcome of assisted reproductive technology [17], and it is also one of the key indicators to choose transfer strategy. After analyzing the age groups, we found that in each age group, the clinical pregnancy rate and the live birth rate was similar between C-2 group and B-1 group. However, there were significant differences in indicators affecting the health of newborns, such as the twin birth rate, preterm birth rate, and low birth weight rate. Therefore, in our study, age has no decisive influence on the selection of embryo transfer strategy.

Interestingly, we found that in different age groups, the incidence of monozygotic twins in the B-1 group was significantly higher than that in the C-2 group. In the past, it was believed that the timing of embryo division governs the ultimate placental configuration of monozygotic twins (MZT) [18]. Some other studies suggested that the splitting of the transferred embryo took place after the blastocyst stage [19]. Although the reason for increased risk of monozygotic twinning in B-1 group is not clear, previous cases reported by others, along with our own report, indicate that blastocyst-stage embryos are more likely to result in monozygosity than cleavage-stage embryos. The reason may be related to the prolongation of embryo culture time in vitro [20–22].

With an increase in age, the clinical pregnancy rate and live birth rate for the four groups had a downward trend. The miscarriage rate increased significantly with the increase in age. However, the birth rate of low birth weight infants in the double-cleavage embryo transfer group was significantly higher compared to the single-blastocyst embryo transfer group for all ages. Therefore, single-blastocyst embryo transfer may be recommended to protect the health of the mother and the baby.

Our study has some limitations, because it is not a randomized controlled study, and only two centers are included. Therefore, we are going to expand the research scope and set up randomized controlled studies in the future, which may provide better guidance in clinical practice.

In summary, single-blastocyst embryo transfer seems to be the best choice for all maternal ages to greatly reduce adverse neonatal outcomes, despite slightly increasing the rate of monozygotic twins and miscarriage, and reducing the rate of live birth. Doctors should select the most suitable transfer strategy based on the patient’s situation. However, with advances in reproductive medicine, single-blastocyst embryo transfer may be the optimal choice for women who desire to get pregnant and deliver a healthy baby.

## Electronic Supplementary Material

ESM 1 (DOCX 46 kb)
