# Supplementary material for: Pregnancy Outcomes of Single/Double Blastocysts and Cleavage Embryo Transfers: a Retrospective Cohort Study of 24,422 Frozen-Thawed Cycles
Source: Reprod Sci. 2020 Aug 25;27(12):2271–8. doi: 10.1007/s43032-020-00247-x (PMC7593290; doi:10.1007/s43032-020-00247-x)
Supplement: Supplementary file 1 — (DOCX 46 kb) [file 43032_2020_247_MOESM1_ESM.docx]

**Supplementary 1 Description and comparison of maternal characteristics, maternal and neonatal outcomes among all of four groups**

|  | **All FET cycles**  **(n=23726)** | **Single cleavage-stage embryo transfer group**  **(C-1)**  **(n=763)** | **Double cleavage-stage embryo transfer group**  **(C-2)**  **(n=13004)** | **Single blastocyst-stage embryo transfer group**  **(B-1)**  **(n=7913)** | **Double blastocyst-stage embryo transfer group**  **(B-2)**  **(n=2046)** | **X^2^** | **P** |
| --- | --- | --- | --- | --- | --- | --- | --- |
| **Maternal Characteristics** |  |  |  |  |  |  |  |
| Maternal age (years) | 32 (29, 35) | 34 (31, 39) | 32 (29, 35) | 32 (29, 35) | 32 (29, 35) |  |  |
| 20-29 | 7269 (30.6) | 129 (16.9) | 4107 (31.6) | 2417 (30.5) | 616 (30.1) | 520.688 | <0.001 |
| 30-34 | 9640 (40.6) | 257 (33.7) | 5289 (40.7) | 3217 (40.7) | 877 (42.9) |  |  |
| 35-37 | 3790 (16.0) | 132 (17.3) | 1956 (15.0) | 1367 (17.3) | 335 (16.4) |  |  |
| 38-39 | 1297 (5.5) | 75 (9.8) | 644 (5.0) | 464 (5.9) | 114 (5.6) |  |  |
| 40-42 | 1084 (4.6) | 71 (9.3) | 603 (4.6) | 329 (4.2) | 81 (4.0) |  |  |
| >42 | 646 (2.7) | 99 (13.0) | 405 (3.1) | 119 (1.5) | 23 (1.1) |  |  |
| Body Mass Index (BMI) | 21.6 (19.8, 24.0) | 21.5 (19.6, 23.9) | 21.5 (19.6, 23.7) | 22.0 (20.0, 24.4) | 21.5 (19.7, 23.8) |  |  |
| Underweight (BMI<18.5) | 1914 (8.1) | 62 (8.1) | 1128 (8.7) | 554 (7.0) | 170 (8.3) | 87.861 | <0.001 |
| Normal weight (18.5≤BMI<24) | 11474 (48.4) | 338 (44.3) | 6259 (48.1) | 3862 (48.8) | 1015 (49.6) |  |  |
| Overweight (24≤BMI<27) | 2940 (12.4) | 84 (11.0) | 1480 (11.4) | 1132 (14.3) | 244 (11.9) |  |  |
| Obesity (BMI≥27) | 1571 (6.6) | 39 (5.1) | 744 (5.7) | 654 (8.3) | 134 (6.5) |  |  |
| Missing value | 5827 (24.6) | 240 (31.5) | 3393 (26.1) | 1711 (21.6) | 483 (23.6) |  |  |
| Infertility type |  |  |  |  |  |  |  |
| Primary infertility | 14463 (61.0) | 402 (52.7) | 8034 (61.8) | 4731 (59.8) | 1296 (63.3) | 33.535 | <0.001 |
| Secondary infertility | 9026 (38.0) | 343 (45.0) | 4828 (37.1) | 3125 (39.5) | 730 (35.7) |  |  |
| Missing value | 237 (1.0) | 18 (2.4) | 142 (1.1) | 57 (0.7) | 20 (1.0) |  |  |
| Cause of infertility |  |  |  |  |  |  |  |
| Tubal | 8730 (36.8) | 259 (33.9) | 5075 (39.0) | 2539 (32.1) | 857 (41.9) | 128.751 | <0.001 |
| Ovulatory | 5575 (23.5) | 201 (26.3) | 3019 (23.2) | 1801 (22.8) | 554 (27.1) | 20.990 | <0.001 |
| Endometriosis | 1455 (6.1) | 65 (8.5) | 835 (6.4) | 440 (5.6) | 115 (5.6) | 14.860 | 0.002 |
| Male factor | 9023 (38.0) | 261 (34.2) | 5025 (38.6) | 3018 (38.1) | 719 (35.1) | 14.081 | 0.003 |
| Unexplained | 5136 (21.6) | 183 (24.0) | 2456 (18.9) | 2138 (27.0) | 359 (17.5) | 215.792 | <0.001 |
| Duration of infertility (years) | 4 (2, 6) | 4 (2, 6) | 4 (2, 6) | 4 (2, 6) | 4 (2, 6) |  |  |
| ≤4 | 14142 (59.6) | 4234 (55.6) | 7964 (61.2) | 4584 (57.9) | 1170 (57.2) | 29.042 | <0.001 |
| >4 | 8957 (37.8) | 308 (40.4) | 4726 (36.3) | 3104 (39.2) | 819 (40.0) |  |  |
| Missing value | 627 (2.6) | 31 (4.1) | 314 (2.4) | 225 (2.8) | 57 (2.8) |  |  |
| Type of fertilization in current cycle |  |  |  |  |  |  |  |
| IVF | 12113 (51.1) | 437 (57.3) | 7074 (54.4) | 3458 (43.7) | 1144 (55.9) | 296.204 | <0.001 |
| ICSI | 10574 (44.6) | 304 (39.9) | 5423 (41.7) | 4075 (51.5) | 771 (37.7) |  |  |
| IVF+ICSI | 1039 (4.4) | 21 (2.8) | 507 (3.9) | 380 (4.8) | 131 (6.4) |  |  |
| Previous ART cycles | 3 (2, 4) | 2 (1, 3) | 1 (1, 2) | 2 (1, 3) | 2 (2, 4) |  |  |
| 0-1 cycles | 10710 (45.1) | 217 (28.5) | 6307 (48.5) | 3727 (47.1) | 458 (22.4) | 768.355 | <0.001 |
| 2-3 cycles | 8590 (36.2) | 328 (43.0) | 4538 (34.9) | 2754 (34.8) | 970 (47.4) |  |  |
| 4-5 cycles | 2185 (9.2) | 85 (11.2) | 923 (7.1) | 831 (10.5) | 346 (16.9) |  |  |
| ≥6 cycles | 804 (3.4) | 49 (6.4) | 299 (2.3) | 317 (4.0) | 139 (6.8) |  |  |
| Missing value | 1437 (6.1) | 83 (10.9) | 936 (7.2) | 285 (3.6) | 133 (6.5) |  |  |
| **Maternal & Neonatal Outcomes** |  |  |  |  |  |  |  |
| Clinical pregnancy | 10237 (43.1) | 164 (21.5) | 5481 (42.1) | 3507 (44.3) | 1085 (53.0) | 237.023 | <0.001 |
| ^*^Monozygotic twins | 151 (1.5) | 3 (1.8) | 47 (0.9) | 79 (2.3) | 22 (2.0) | 31.668^@^ | <0.001^@^ |
| ^*^Miscarriage | 1687 (16.5) | 39 (23.8) | 786 (14.3) | 632 (18.0) | 230 (21.2) | 48.180 | <0.001 |
| ^*^Maternal complication | 968 (9.5) | 12 (7.1) | 515 (9.4) | 312 (8.9) | 129 (11.9) | 9.686 | 0.021 |
| Live birth | 6368 (26.8) | 90 (11.8) | 3771 (29.0) | 1819 (23.0) | 688 (33.6) | 226.611 | <0.001 |
| ^∆^Twin birth | 1116 (17.5) | 0 (0.0) | 883 (23.4) | 34 (1.9) | 199 (28.9) | 479.969 | <0.001 |
| ^∆^Preterm birth | 936 (14.7) | 6 (6.7) | 609 (16.1) | 175 (9.6) | 146 (21.2) | 71.715 | <0.001 |
| ^∆^Low birth weight | 776 (12.2) | 3 (3.3) | 559 (14.8) | 92 (5.1) | 122 (17.7) | 137.261 | <0.001 |
| ^∆^SGA | 362 (5.7) | 4 (4.4) | 267 (7.1) | 55 (3.0) | 36 (5.2) | 38.245 | <0.001 |

Notes: C-1 indicates the single cleavage-stage embryo transfer group; C-2 indicates the double cleavage-stage embryo transfer group; B-1 indicates the single blastocyst-stage embryo transfer group; B-2 indicates the double blastocyst-stage embryo transfer group. Values are n (%) or median (1st quartile, 3rd quartile). ^*^ The denominator is the number of clinical pregnancies in each group. ^∆^ The denominator is the number of live births in each group. ^@^ Likelihood Ratio for the case that one or more cells has expected count less than 5.

**Supplementary 2 Pairwise comparisons of maternal characteristics, maternal and neonatal outcomes between groups**

|  | **C1 vs C2** | **C1 vs B1** | **C1 vs B2** | **C2 vs B1** | **C2 vs B2** | **B1 vs B2** |
| --- | --- | --- | --- | --- | --- | --- |
| **Maternal Characteristics** | |  |  |  |  |  |
| Maternal age  (20-29; 30-34; 35-37; 38-39; 40-42; >42) | **X^2^=317.939**  **P<0.001** | **X^2^=474.611**  **P<0.001** | **X^2^=265.949**  **P<0.001** | **X^2^=77.405**  **P<0.001** | **X^2^=29.946**  **P<0.001** | X^2^=3.526  P=0.474 |
| Body Mass Index (BMI)  (Underweight; Normal weight; Overweight; Obesity) | X^2^=0.217  P=0.975 | X^2^=10.669  P=0.014 | X^2^=0.982  P=0.806 | **X^2^=84.753**  **P<0.001** | X^2^=2.087  P=0.555 | **X^2^=16.172**  **P=0.001** |
| Infertility type  (Primary; Secondary) | **X^2^=21.612**  **P<0.001** | **X^2^=11.088**  **P=0.001** | **X^2^=22.996**  **P<0.001** | **X^2^=10.361**  **P=0.001** | X^2^=1.695  P=0.193 | **X^2^=9.504**  **P=0.002** |
| Cause of infertility |  |  |  |  |  |  |
| Tubal | **X^2^=7.841**  **P=0.005** | X^2^=1.100  P=0.294 | **X^2^=14.638**  **P<0.001** | **X^2^=102.347**  **P<0.001** | X^2^=6.056  P=0.014 | **X^2^=69.480**  **P<0.001** |
| Ovulatory | X^2^=3.934  P=0.047 | X^2^=5.034  P=0.025 | X^2^=0.152  P=0.696 | X^2^=0.577  P=0.448 | **X^2^=14.559**  **P<0.001** | **X^2^=16.782**  **P<0.001** |
| Endometriosis | X^2^=5.191  P=0.023 | **X^2^=11.111**  **P=0.001** | **X^2^=7.784**  **P=0.005** | X^2^=6.366  P=0.012 | X^2^=1.915  P=0.166 | X^2^=0.011  P=0.916 |
| Male factor | X^2^=5.993  P=0.014 | X^2^=4.578  P=0.032 | X^2^=0.214  P=0.644 | X^2^=0.524  P=0.469 | **X^2^=9.178**  **P=0.002** | X^2^=6.233  P=0.013 |
| Unexplained | **X^2^=12.088**  **P=0.001** | X^2^=3.270  P=0.071 | **X^2^=14.791**  **P<0.001** | **X^2^=189.827**  **P<0.001** | X^2^=2.088  P=0.148 | **X^2^=77.644**  **P<0.001** |
| Duration of infertility  (≤4years; >4years) | X^2^=6.901  P=0.009 | X^2^=0.803  P=0.370 | X^2^=0.179  P=0.673 | **X^2^=19.858**  **P<0.001** | **X^2^=11.325**  **P=0.001** | X^2^=0.422  P=0.516 |
| Type of fertilization in current cycle  (IVF; ICSI; IVF+ICSI) | X^2^=4.165  P=0.125 | **X^2^=53.672**  **P<0.001** | **X^2^=14.554**  **P=0.001** | **X^2^=225.238**  **P<0.001** | **X^2^=33.829**  **P<0.001** | **X^2^=124.352**  **P<0.001** |
| Previous ART cycles  (0-1; 2-3; 4-5; ≥6) | **X^2^=143.214**  **P<0.001** | **X^2^=77.292**  **P<0.001** | **X^2^=22.292**  **P<0.001** | **X^2^=110.829**  **P<0.001** | **X^2^=664.703**  **P<0.001** | **X^2^=395.177**  **P<0.001** |
| **Maternal & Neonatal Outcomes** | |  |  |  |  |  |
| Clinical pregnancy | **X^2^=127.099**  **P<0.001** | **X^2^=148.536**  **P<0.001** | **X^2^=223.828**  **P<0.001** | **X^2^=9.461**  **P=0.002** | **X^2^=85.116**  **P<0.001** | **X^2^=49.642**  **P<0.001** |
| ^*^Monozygotic twins | X^2^=1.322^@^  P=0.250^@^ | X^2^=0.137^@^  P=0.712^@^ | X^2^=0.029^@^  P=0.864^@^ | **X^2^=30.115**  **P<0.001** | **X^2^=11.926**  **P=0.001** | X^2^=0.195  P=0.659 |
| ^*^Miscarriage | **X^2^=11.371**  **P=0.001** | X^2^=3.479  P=0.062 | X^2^=0.562  P=0.453 | **X^2^=21.804**  **P<0.001** | **X^2^=32.566**  **P<0.001** | X^2^=5.485  P=0.019 |
| ^*^Maternal complication | X^2^=0.813  P=0.367 | X^2^=0.486  P=0.486 | X^2^=2.974  P=0.085 | X^2^=0.639  P=0.424 | X^2^=6.365  P=0.012 | **X^2^=8.550**  **P=0.003** |
| Live birth | **X^2^=105.697**  **P<0.001** | **X^2^=50.792**  **P<0.001** | **X^2^=132.264**  **P<0.001** | **X^2^=90.778**  **P<0.001** | **X^2^=18.159**  **P<0.001** | **X^2^=97.689**  **P<0.001** |
| ^∆^Twin birth | **X^2^=27.323**  **P<0.001** | X^2^=0.811^@^  P=0.368^@^ | **X^2^=34.979**  **P<0.001** | **X^2^=415.417**  **P<0.001** | **X^2^=9.608**  **P=0.002** | **X^2^=433.444**  **P<0.001** |
| ^∆^Preterm birth | X^2^=5.903  P=0.015 | X^2^=0.872  P=0.350 | **X^2^=10.724**  **P=0.001** | **X^2^=43.379**  **P<0.001** | **X^2^=10.639**  **P=0.001** | **X^2^=60.167**  **P<0.001** |
| ^∆^Low birth weight | **X^2^=9.331**  **P=0.002** | X^2^=0.236^@^  P=0.627^@^ | **X^2^=12.237**  **P<0.001** | **X^2^=113.739**  **P<0.001** | X^2^=3.805  P=0.051 | **X^2^=102.717**  **P<0.001** |
| ^∆^Congenital malformation | X^2^=0.132^@^  P=0.717^@^ | X^2^=0.092^@^  P=0.761^@^ | X^2^=0.354^@^  P=0.552^@^ | X^2^=0.005  P=0.943 | X^2^=0.151^@^  P=0.697^@^ | X^2^=0.610^@^  P=0.721^@^ |

Notes: C-1 indicates the single cleavage-stage embryo transfer group; C-2 indicates the double cleavage-stage embryo transfer group; B-1 indicates the single blastocyst-stage embryo transfer group; B-2 indicates the double blastocyst-stage embryo transfer group. ^*^ The denominator is the number of clinical pregnancies in each group. ^∆^ The denominator is the number of live births in each group. ^@^ Likelihood Ratio for the case that one or more cells has expected count less than 5. **BOLD** in X^2^ values and P values indicates P<0.008 (the Bonferroni-corrected p-value for multiple pairwise comparisons).

**Supplementary 3 Predicted absolute risks (probabilities) of each maternal outcome in each maternal age group**

| **Maternal age (years)** |  | **Clinical pregnancy** | |  | **^*^Monozygotic twins** | |  | **^*^Miscarriage** | |  | **^*^Maternal complication** | |
| --- | --- | --- | --- | --- | --- | --- | --- | --- | --- | --- | --- | --- |
|  |  | **C-2** | **B-1** |  | **C-2** | **B-1** |  | **C-2** | **B-1** |  | **C-2** | **B-1** |
| 20-29 |  | 0.5035 | 0.5126 |  | 0.0083 | 0.0233 |  | 0.1066 | 0.1315 |  | 0.0869 | 0.0776 |
| 30-34 |  | 0.4393 | 0.4487 |  | 0.0070 | 0.0199 |  | 0.1296 | 0.1585 |  | 0.1043 | 0.0945 |
| 35-37 |  | 0.3772 | 0.3840 |  | 0.0060 | 0.0169 |  | 0.1595 | 0.1906 |  | 0.1256 | 0.1129 |
| 38-39 |  | 0.3172 | 0.3280 |  | 0.0051 | 0.0147 |  | 0.1921 | 0.2305 |  | 0.1467 | 0.1353 |
| 40-42 |  | 0.2649 | 0.2729 |  | 0.0043 | 0.0123 |  | 0.2327 | 0.2787 |  | 0.1619 | 0.1539 |
| >42 |  | 0.2153 | 0.2178 |  | 0.0047 | 0.0111 |  | 0.3097 | 0.3392 |  | 0.2041 | 0.1640 |

Notes: C-2 indicates the double cleavage-stage embryo transfer group; B-1 indicates the single blastocyst-stage embryo transfer group. ^*^ The denominator is the number of clinical pregnancies in each group. The probabilities of each maternal or neonatal outcome in each maternal age group were calculated by using the model of multivariable logistic regressions.

**Supplementary 4 Predicted absolute risks (probabilities) of each neonatal outcome in each maternal age group**

| **Maternal age (years)** |  | **Live birth** | |  | **^∆^Twin birth** | |  | **^∆^Preterm birth** | |  | **^∆^Low birth weight** | |  | **^∆^Congenital malformation** | |
| --- | --- | --- | --- | --- | --- | --- | --- | --- | --- | --- | --- | --- | --- | --- | --- |
|  |  | **C-2** | **B-1** |  | **C-2** | **B-1** |  | **C-2** | **B-1** |  | **C-2** | **B-1** |  | **C-2** | **B-1** |
| 20-29 |  | 0.2993 | 0.254 |  | 0.2781 | 0.0222 |  | 0.1776 | 0.1001 |  | 0.1464 | 0.0479 |  | 0.0029 | 0.0041 |
| 30-34 |  | 0.2528 | 0.2134 |  | 0.2254 | 0.0171 |  | 0.1619 | 0.0907 |  | 0.1353 | 0.0442 |  | 0.0032 | 0.0051 |
| 35-37 |  | 0.2098 | 0.1726 |  | 0.1845 | 0.0127 |  | 0.1569 | 0.0865 |  | 0.1338 | 0.041 |  | 0.0034 | 0.0054 |
| 38-39 |  | 0.1696 | 0.1443 |  | 0.1484 | 0.0106 |  | 0.1378 | 0.0834 |  | 0.1224 | 0.0412 |  | 0.0025 | 0.0053 |
| 40-42 |  | 0.1401 | 0.1132 |  | 0.1276 | 0.0083 |  | 0.1433 | 0.0744 |  | 0.1289 | 0.0371 |  | 0.0035 | 0.0035 |
| >42 |  | 0.1107 | 0.0842 |  | 0.1058 | 0.0072 |  | 0.1742 | 0.0798 |  | 0.1387 | 0.0446 |  | 0.0081 | 0.0024 |

Notes: C-2 indicates the double cleavage-stage embryo transfer group; B-1 indicates the single blastocyst-stage embryo transfer group. ^∆^ The denominator is the number of live births in each group. The probabilities of each maternal or neonatal outcome in each maternal age group were calculated by using the model of multivariable logistic regressions.
